# RETRACTION: Effect and Mechanism of Total Flavonoids Extracted from *Cotinus Coggygria* against Glioblastoma Cancer *in Vitro* and *in Vivo*

**DOI:** 10.1155/bmri/9857978

**Published:** 2025-12-18

**Authors:** BioMed Research International

RETRACTION: G. Wang, J.J. Wang, L. Du, F. Li, “Effect and Mechanism of Total Flavonoids Extracted from *Cotinus coggygria* against Glioblastoma Cancer *In Vitro* and *In Vivo*,” *BioMed Research International*, 2015, 856349, https://doi.org/10.1155/2015/856349.

The above article, published online on 18 October 2015 in Wiley Online Library (http://wileyonlinelibrary.com), has been retracted by agreement between the journal Associate Editor, Jinsong Ren, and John Wiley & Sons Ltd. The retraction has been agreed due to errors identified in the figures, also raised on PubPeer [1]. The authors requested corrections to Figures 4 and 5, however subsequent investigation also identified inconsistencies in Figure 2. A summary of these concerns is as follows:

• The DBTRG‐05MG + CCF 100 *μ*g/mL panel in Figure 2 appears identical to the 100 *μ*g/mL panel in Figure 3a of [2]
• The U251 + CCF 50 *μ*g/mL panel in Figure 2 appears identical to the 50 *μ*g/mL panel in Figure 3b of [2]
• The U251 + CCF 100 *μ*g/mL panel in Figure 2 appears identical to the 100 *μ*g/mL panel in Figure 3b of [2]
• The DBTRG‐05MG + CCF 50 *μ*g/mL panel in Figure 2 appears identical to the 50 *μ*g/mL panel in Figure 3a of [2]
• The DBTRG‐05MG + DMSO panel in Figure 2 appears identical to the DMSO panel in Figure 3c of [2]
• In Figure 4a, the U87 25, 50 and 100 *μ*g/mL plots appear similar to the 50, 100 and 200 *μ*M plots shown in Figure 3b of [3]
• In Figure 4a, the U251/DMSO plot appears similar to the MYR/DMSO plot in Figure 3b of [4]
• In Figure 4a, the DBTRG‐05MG/DMSO panel appears similar to the MYR‐NLs/Control‐NLs panel in Figure 3b of [4]
• The *β*‐actin bands in Figure 4b appear identical to the *β*‐actin bands in Figure 9a and 10c of [2]
• In Figure 5a, the Akt, p‐Akt (Ser 473) and *β*‐actin bands appear identical to the Akt, p‐Akt (Ser 473) and *β*‐actin bands shown in Figure 3a of [5]

After assessment by the editorial board, the errors render the results and conclusions unreliable and therefore the article is retracted.

The authors did not respond to the retraction.
